# Extra-osseous Roles of the RANK-RANKL-OPG Axis with a Focus on Skeletal Muscle

**DOI:** 10.1007/s11914-024-00890-2

**Published:** 2024-09-26

**Authors:** John Gostage, Paul Kostenuik, Katarzyna Goljanek-Whysall, Ilaria Bellantuono, Eugene McCloskey, Nicolas Bonnet

**Affiliations:** 1 The Medical Research Council/Versus Arthritis Centre for Integrated Research Into Musculoskeletal Aging, CIMA, University of Liverpool, Liverpool, UK; 2 Division of Clinical Medicine, School of Medicine and Population Health, Healthy Lifespan Institute and the Centre for Integrated Research in Musculoskeletal Aging, University of Sheffield, Sheffield, UK; 3 Discipline of Physiology, School of Medicine, University of Galway, Galway, Ireland; 4 School of Dentistry and Phylon Pharma Services, University of Michigan, Thousand Oaks, CA USA; 5 Service of Bone Diseases, Department of Medicine, Geneva University Hospital and Faculty of Medicine, Geneva, Switzerland

**Keywords:** Osteoprotegerin, Denosumab, RANK-RANKL-OPG axis, NF-κB signalling, Skeletal muscle, Extra-osseous

## Abstract

**Purpose of Review:**

This review aims to consolidate recent observations regarding extra-osseous roles of the RANK-RANKL-OPG axis, primarily within skeletal muscle.

**Recent Findings:**

Preclinical efforts to decipher a common signalling pathway that links the synchronous decline in bone and muscle health in ageing and disease disclosed a potential role of the RANK-RANKL-OPG axis in skeletal muscle. Evidence suggests RANKL inhibition benefits skeletal muscle function, mass, fibre-type switching, calcium homeostasis and reduces fall incidence. However, there still exists ambiguity regarding the exact mechanistic actions and subsequent functional improvements. Other potential RANK-RANKL-OPG extra-osseous roles include regulation of neural-inflammation and glucose metabolism.

**Summary:**

Growing evidence suggests the RANK-RANKL-OPG axis may play a regulatory role in extra-osseous tissues, especially in skeletal muscle. Targeting RANKL may be a novel therapy in ameliorating loss of muscle mass and function. More research is warranted to determine the causality of the RANK-RANKL-OPG axis in extra-osseous tissues, especially those affected by aging.

## Introduction

Receptor activator of nuclear factor kappa B (RANK), RANK ligand (RANKL), and osteoprotegerin (OPG) comprise the RANK-RANKL-OPG axis, a complex, and multifaceted signalling system. The axis has a pivotal role in regulating bone mass and calcium homeostasis by controlling osteoclasts and bone remodelling [1, 2]. The axis also has other prominent roles, including, but not restricted to, endothelial physiology, angiogenesis, cell proliferation [3], inflammation, immunity [4, 5], tumorigenesis, metastasis [6] and brain signalling [7, 8]. RANKL specifically binds to and activates RANK in a manner that is inhibited when RANKL first binds to OPG, a soluble decoy receptor of RANKL. RANKL can either be cell membrane-bound (M-RANKL) or soluble (S-RANKL). Given its location, M-RANKL can also participate in “reverse” signalling [9]. In recent years, it has been suggested that RANKL and OPG are osteokines that are involved in bone and extra-osseous tissue crosstalk, with close anatomical neighbours, skeletal muscles, being potential targets [10].

Binding of RANKL by OPG inhibits osteoclastogenesis which suggested a potential therapeutic role for OPG in diseases of bone loss such as osteoporosis or cancer. OPG has various domains including an N-terminal cysteine rich region which is the primary site for RANKL binding, a death domain homologous region which can interact with TNF related apoptosis inducing ligand (TRAIL), and a C-terminal heparin-binding domain which allows for interaction with, for example, heparan sulfates and integrins. The latter domain limits the circulating half-life of OPG, and deletion of this domain was among the early strategies in creating recombinant therapeutic versions of OPG for infrequent administration [11]. Concurrent efforts pursued the production of fully human therapeutic antibodies to RANKL, which have even longer half-lives than optimized versions of OPG and do not bind TRAIL [12]. The first such anti-RANKL to enter clinical development was originally named AMG162 (subsequently called denosumab). A pivotal Phase 3 clinical trial (FREEDOM) in women with postmenopausal osteoporosis (PMO) showed that denosumab significantly increased bone mineral density (BMD) at the hip, femoral neck, lumbar spine, and distal radius and reduced the risk of vertebral, nonvertebral, and hip fractures [13]. Numerous other trials confirm the ability of denosumab to increase BMD and reduce the risk of fragility fractures (reviewed in [14]). Interestingly, in FREEDOM, denosumab was associated with a significantly lower incidence of falls and fall-related concussions, suggesting potential extra-osseous benefits of RANKL inhibition including improved skeletal muscle function [13].

An early study to explore a potential effect of the RANK-RANKL-OPG pathway in muscle was undertaken in muscular dystrophy (*mdx* mice) as this model exhibited severe muscle degeneration alongside low BMD. While OPG, administered as OPG-Immunoglobulin Fc segment complex (OPG-Fc), had no effect on wildtype (WT) mice muscle force, OPG-Fc rescued muscle force in a dose-dependent manner in the soleus, extensor digitorum longus (EDL) and diaphragm muscles of *mdx* mice [15]. However, this observation brought with it many new questions; for example, is this muscle effect mediated by OPG inhibition of RANKL or a RANKL-independent mechanism, are the benefits limited to dystrophic or severely degenerated muscle, and is this a direct effect on muscle or mediated by bone-to-muscle crosstalk? This review aims to highlight some of the notable findings of studies of the RANK-RANKL-OPG axis in skeletal muscle with additional reference to effects in other extra-osseous tissues.

## Observed Effects of RANK-RANKL-OPG Modifications on Muscle Mass and Performance

### Clinical Observations

Following on from the FREEDOM falls observation, a meta-analysis investigated fall risk across five placebo-controlled trials of denosumab [16], three associated with osteoporosis  [13, 17, 18] and two associated with cancer treatment-induced bone loss [19, 20]. Prior to all studies, participants received no specific advice about exercise or falls prevention. Meta-analysis showed that the Kaplan–Meier estimated incidence of falls in the pooled placebo groups was 6.5%, compared with 5.2% in denosumab groups (a 21% reduction, *p* = 0.0061). A greater reduction in falls was observed with denosumab treatment in subjects aged < 75 years versus subjects ≥ 75 years (hazard ratios 0.65, 95% CI 0.52–0.82 and 1.01, 95% CI 0.78–1.31, respectively), potentially reflecting the impact of other factors on falls risk in the oldest old (multimorbidity, frailty, and other deficits, e.g., polypharmacy, orthostatic hypotension, vestibular disorders, cataracts, and macular degeneration) (Table 1) [16].

**Table 1:** Clinical studies exploring the potential roles of the RANK-RANKL-OPG axis on skeletal muscle mass and performance and fall incidence. Table organised in chronological order

Title, author, date, reference	Study parameters	Major findings
Denosumab for prevention of fractures in postmenopausal women with osteoporosis Cummings *et al*., 2009 [13]	Postmenopausal women were treated with either placebo (*N* = 3906) or denosumab (DMab) 60mg (*N* = 3902) every 6 months for 3 years	** > **Denosumab treatment for 3 years was associated with a significant reduction in falls (adverse events), when compared to placebo group (*p* = 0.02)
Treatment of facioscapulohumeral muscular dystrophy with Denosumab Lefkowitz, Lefkowitz and Kethley, 2012 [24]	Case study: 66-year-old female (diagnosed with facioscapulohumeral muscular dystrophy and osteoporosis). Treated with DMab 60mg. Second injection given 63 days after the first. A total of seven injections were given (with 40–42-day intervals post second injection)	** > **Drastic reversal of dystrophic symptoms was observed after every DMab injection. Reversal of beneficial effects initiated around 6–7 weeks post DMab injection ** > **24 h after 1st injection: Subject was able to walk without a cane, bury her eyelashes, swallow easier, open bottles (was unable to do prior to the DMab injection), and was physically stronger and exhibited improved balance ** > **24 h after 2nd injection: Subject was able to whistle, walk in high heels, snap her fingers, and play the piano without fatigue (amongst other, not listed improvements). Significant improvements in hand grip strength, get up and go test and sit to stand test ** > **24 h after 3rd injection: Further significant improvements in hand grip strength, get up and go test and sit to stand test
RANKL inhibition improves muscle strength and insulin sensitivity and restores bone mass. Bonnet *et al*., 2019 [22]	PMO women treated with DMab (*N* = 18, 65 ± 1.5 years), bisphosphonates (zoledronate *N* = 12, alendronate *N* = 8, 65.7 ± 0.9 years) or vehicle (*N* = 55, 65 ± 1.4 years). The groups were matched for age, BMI, BMD and fracture prevalence at baseline	** > **In PMO women, 3 years of DMab treatment improved appendicular lean mass and handgrip strength compared to no treatment, whereas bisphosphonates did not ** > **Muscle parameters strongly correlated with changes in lumbar spine BMD
Effect of Denosumab on Falls, Muscle Strength, and Function in Community‐Dwelling Older Adults. Phu *et al*., 2019 [21]	Longitudinal study (6 month) on the effects of DMab (*N* = 51) or zoldronate (*N* = 28) on muscle function of community-dwelling older adults	** > **DMab significantly improved gait speed whilst also enhancing multidirectional agility ** > **Similar effects were seen in subjects receiving zoledronate, but DMab treatment was associated with greater improvements in the four-square step test and in a subjective measure for fear of falling
A Pooled Analysis of Fall Incidence From Placebo-controlled Trials of Denosumab Chotiyarnwong *et al*., 2020 [16]	Meta-analysis of 5 placebo-controlled trials of DMab. Pooled characteristics: placebo *N* = 5006, 71.9 ± 6.9 years; DMab *N* = 5030, 71.8 ± 6.8 years (83.1% female)	** > **Estimated Kaplan–Meier incidence of falls was significantly higher in the placebo groups (6.5%), compared to DMab groups (5.2%) (*p* = 0.0061) ** > **DMab treated subjects aged < 75 years were 35% less likely to fall versus matched controls
Is there a potential dual effect of denosumab for treatment of osteoporosis and sarcopenia? Miedany *et al*., 2021 [33]	A longitudinal multicenter, controlled, prospective study. Study group: DMab (primary or combinatorial) treatment for postmenopausal/senile osteoporosis (*N* = 135, 5 years). Control group: treat-to-target, including zoledronate (*N* = 136, 3 years) and alendronate (*N* = 136, 5 years). 1st osteoporotic and sarcopenic assessment after 3–5 years of treatment, 2nd assessment after 1 year stopping osteoporosis therapy	** > **Upon completing DMab therapy (5 years), there was significant decrease in falls risk (*p* = 0.001) and significant improvements in all sarcopenia measures (grip strength, timed up and go and 4m walk, *p* = 0.01). One-year post-discontinuation of DMab, a significant worsening of both sarcopenia measures and falls risk (*p* = 0.01) was noted
Beneficial effects of denosumab on muscle performance in patients with low BMD: a retrospective, propensity score-matched study. Rupp *et al*., 2022 [23]	Retrospective, propensity score-matched (sex, age, BMI, follow-up time) cohort study. 150 patients with osteopenia or osteoporosis receiving vitamin D (*N* = 60), bisphosphonates (*N* = 30) or DMab (*N* = 60) therapy. Mean follow up: 17.6 ± 9 months	** > **DMab (*p* < 0.001) and bisphosphonate (*p* = 0.001) treatments significantly increased grip force compared to vitamin D. DMab group significantly increased chair rising test force compared to the bisphosphonate group. Neither the changes in bone metabolic parameters nor BMD were associated with changes in muscle performance
RANKL Blockade Reduces Cachexia and Bone Loss Induced by Non‐Metastatic Ovarian Cancer in Mice. Pin *et al*., 2022 [34]	Female adults with ovarian cancer assessed (cachexia *N* = 8, control *N* = 19)	** > **Human ovarian cancer is associated with elevated RANKL, cachexia, and bone loss

Outside the setting of randomised control trials, limited comparative data support potential benefits of denosumab on skeletal muscle (Table 1). In a non-randomised study of a clinical cohort, denosumab treatment (*n* = 51) significantly improved gait speed, timed get up and go test and four-square step test over 6 months, in community-dwelling older adults [21] (Table 1). In another non-randomised cohort study, 18 PMO women treated for osteoporosis with denosumab over 3 years were compared to 55 controls and 20 patients treated by bisphosphonate. Denosumab treatment was associated with increased appendicular lean mass (ALM) and hand grip strength compared with baseline [22] (Table 1). In a retrospective, propensity score-matched (sex, age, BMI, follow-up time) cohort study, both denosumab and bisphosphonate treatment were associated with a higher increase in grip force than a comparator group receiving vitamin D only. Those treated with denosumab also showed a greater improvement in chair rising test force compared to the bisphosphonates group [23] (Table 1). Interestingly, in a case study of facioscapulohumeral muscular dystrophy, a switch from Forteo (teriparatide, 2 years of treatment) to denosumab to help combat worsening osteoporosis resulted in a rapid reversal of many dystrophic symptoms (Table 1) [24].

Additionally, muscle loss has been associated with Paget’s disease (OPG deficiency) [25, 26]. Interestingly, Juvenile Paget’s disease of bone was also shown to be associated with retinal abnormalities, including peripapillary atrophy, angioid streaks, and choroidal neovascularization [27]. Recently, ocular manifestations were also identified in a novel duplication variant of TNFRSF11A (RANK), in familial Paget’s disease [28]. Taken together the RANK-RANKL-OPG axis may also be related to ocular health, with a potential modality being vascular maintenance – OPG activity has been associated with peripheral artery disease, vascular calcification and atherosclerosis, and abdominal aortic aneurysms [29–32].

### Preclinical Observations

*In vitro* (Table 2) and *in vivo* models (Table 3) have been useful in elucidating the causality of RANK-RANKL-OPG signalling in skeletal muscle. OPG knockout mice (*Opg*^*−/−*^), expressing higher levels of circulating RANKL exhibited notable changes in skeletal muscle mass and function, including markedly reduced EDL weight and force production, lower activity levels, and whole limb grip force. Furthermore, *Opg*^*−/−*^ mice treated with anti-RANKL IK22-5 were able to walk or run farther distances and exhibited significant improvements in whole limb grip force and EDL maximum specific force [35]. Denervated mice lacking skeletal muscle RANK (*RANK*^*mko*^) exhibited reduced EDL muscle mass and specific force versus genotype and treatment controls [36] (Table 3).

**Table 2:** Utilisation of *in vitro* models to explore the potential role of the RANK-RANKL-OPG axis in skeletal muscle in a preclinical setting. Table organised in chronological order

Title, author, date, reference	Study parameters	Major findings
Osteoprotegerin protects against muscular dystrophy Dufresne *et al*., 2015 [15]	Determining OPG and RANKL release from LPS (1 μg/ml) stimulated C2C12 myotubes. 5-day differentiation protocol	** > **LPS stimulated myotube OPG release (significant increase after 16 h, ~ 800 pg/ml) ** > **S-RANKL protein was undetectable in the C2C12 culture media after LPS stimulation
Muscle RANK is a key regulator of Ca^2+^ storage, SERCA activity, and function of fast-twitch skeletal muscles. Dufresne *et al*., 2016 [36]	Characterising C2C12 myotubes (after 5 days of differentiation)	** > **RANK was undetectable in proliferating C2C12 myoblasts ** > **C2C12 myotubes express RANK
Angiogenin and Osteoprotegerin are type II muscle specific myokines protecting pancreatic beta-cells against pro-inflammatory cytokines. Rutti *et al*., 2018 [43]	Characterising primary human myoblasts sourced from either the soleus or triceps brachii (donors age: 22.79 ± 0.38), and the effects of conditioned media on beta cells	** > **Cultured differentiated primary human triceps brachii skeletal muscle cells secreted significantly more OPG compared to soleus muscle cells. 24 h of 20ng/ml TNF-α exposure significantly increased OPG levels in human triceps myotubes, compared to no TNF-α treatment ** > **OPG counteracts the negative effects of conditioned medium from soleus skeletal muscle cells on primary pancreatic beta-cells proliferation and insulin secretion
RANKL inhibition improves muscle strength and insulin sensitivity and restores bone mass. Bonnet *et al*., 2019 [22]	C2C12s ± chronic RANKL ± OPG-Fc	** > **In C2C12s, OPG-Fc reversed the effects of chronic RANKL exposure by blocking NF-κB signalling (as shown by reduced Fos, Jun and NFAT expression) ** > **Chronic insulin exposure plus OPG-Fc treatment to C2C12s increased Glut-1, Glut-4, and Fabp4 gene expression, limited the phosphorylation of IRS1-Ser318 and decreased glucose levels (compared to chronic insulin exposure alone)
Muscle weakness and selective muscle atrophy in osteoprotegerin-deficient mice. Hamoudi *et al*., 2020 [35]	C2C12 myotubes ± RANKL 100 ng/ml (for up to 48 h)	** > **C2C12 myotubes exposed to RANKL had significantly lower myotube CSA and significantly increased p-NFκB-p65 (after 15 min), Atrogin-1 (after 1 h) and MuRF-1 (after 6 h) protein expression
Testing the efficacy of a human full-length OPG-Fc analog in a severe model of cardiotoxin-induced skeletal muscle injury and repair. Bouredji *et al*., 2021 [37]	C2C12s ± hFL-OPG-Fc 50–1000 ng/ml (4 days of differentiation, followed by 24-72 h of treatment). For CTX investigations, cells were exposed to CTX (1 μM) for 1 h	** > **200–1000 ng/ml hFL-OPG-Fc significantly increased C2C12 fiber diameter (after 24 h) and 1000 ng/ml significantly increased C2C12 fusion index (after 72 h) ** > **hFL-OPG-Fc treatment attenuated CTX-induced cytotoxicity *in vitro*, as shown by a reduction in lactate dehydrogenase, creatine kinase, cleaved-caspase 3, Bax and TUNEL^+^ apoptotic cells, and an increase in β3 integrin
RANKL Mediates Muscle Atrophy and Dysfunction in a Cigarette Smoke-induced Model of Chronic Obstructive Pulmonary Disease. Xiong *et al*., 2021 [38]	C2C12s ± RANK-siRNA ± recombinant murine RANKL 10–200 ng/ml. 5-day differentiation protocol, treatment times varied between 30 min – 48 h	** > **In C2C12 myotubes, RANKL treatment significantly reduced, viability, FoxO3a ratio and MyHC expression and significantly increased the NF-κB-p65 ratio and Atrogin-1 and MuRF1 ** > **RANK-siRNA kept Atrogin-1, MuRF1 and NF-κB-p65 ratio levels close to baseline in C2C12 myotubes, and significantly reduced the expression levels of these when treated with RANKL
RANKL Blockade Reduces Cachexia and Bone Loss Induced by Non‐Metastatic Ovarian Cancer in Mice. Pin *et al*., 2022 [34]	C2C12 and human skeletal muscle myotubes ± RANKL (200 ng/ml) for 48 h. C2C12 myotubes and ES-2 co-culture ± anti-RANKL (CD254) for 48 h. 5-day differentiation protocol	** > **RANKL treatment significantly reduced C2C12 and human myotube fiber diameter ** > **Anti-RANKL reduced C2C12 myotube atrophy induced by ES-2 (ovary cancer) cells
Delayed denervation-induced muscle atrophy in Opg knockout mice. Zhang *et al*., 2023 [41]	Characterisation of isolated satellite cells (± differentiation protocols) from *Opg* ^*−/−*^ and WT 6-week-old male mice	** > **Satellite cells isolated from *Opg *^*−/−*^ mice exhibited reduced proliferation but increased differentiation index and fusion index once differentiated for 48 h (when compared to WT) ** > **Tet2 expression increased in differentiated satellite cells isolated from *Opg *^*−/−*^ mice (when compared to WT)
RANKL signaling drives skeletal muscle into the oxidative profile. Cavalcanti de Araújo *et al*., 2024 [42]	Mitochondrial function was explored in C2C12 myotubes ± 20 ng/ml RANKL. 5-day differentiation protocol	** > **After 15 min of RANKL exposure, C2C12 myotubes showed significant increases in phospho-ERK/total ERK and phospho-p38/total p38 ratios ** > **RANKL treatment (24 h) significantly increased mitochondrial area, mtDNA/nDNA ratio and expression of citrate synthase, ATP-synthase, and NRF-1, a p38 MAPK inhibitor (SB203580) and MEK inhibitor (U0126) ameliorated these effects ** > **C2C12 myotubes exhibited significant reductions in PTPRG and MuRF1 gene expression and increased spare respiratory capacity after 24 h of RANKL treatment

**Table 3:** Utilisation of *in vivo* models to explore the potential role of the RANK-RANKL-OPG axis in skeletal muscle in a preclinical setting. * denotes studies in a dystrophic setting. Table organised in chronological order

Title, author, date, reference	Study parameters	Major findings
Systemic cytokine response following exercise-induced muscle damage in humans Philippou *et al*., 2009 [44]	Young males (*N* = 10) performed quadricep eccentric exercises. Blood samples were obtained before exercise and 6 h, 2 days, 5 days and 16 days post-exercise	** > **Up to 2 days post exercise OPG and IL-6 concentrations were increased, and RANKL was decreased. Indication of a common modulating role of IL-6 and the OPG/RANKL system during skeletal muscle regeneration following damage
*Osteoprotegerin protects against muscular dystrophy Dufresne *et al*., 2015 [15]	Assessment of *mdx* dystrophic mice (C57BL/10ScSn-Dmdmdx/J) – daily intraperitoneal injection of PBS or OPG-Fc (0.3 or 1 mg/kg/day) for 10 days (days 25 to 35 after birth)	** > **0.3 mg/kg OPG-Fc significantly increased maximal specific force of *mdx* soleus (46%) and mdx EDL (114%) ** > **1 mg/kg OPG-Fc significantly increased maximal specific force of *mdx* soleus (70%), *mdx* EDL (223%) and mdx diaphragm (59%) muscles, and reduced muscle damage and macrophage (47%) and neutrophil (68%) infiltration of *mdx* EDL muscles
Muscle RANK is a key regulator of Ca^2+^ storage, SERCA activity, and function of fast-twitch skeletal muscles. Dufresne *et al*., 2016 [36]	Muscle mass and functionality of *RANK*^*mko*^ mice (specific RANK skeletal muscle deletion). Sciatic denervation was performed on adult mice aged 12-18 weeks.	**> **Denervated EDL muscles from *RANK*^*mko*^ mice exhibited reduced mass, almost absent slow-twitch fibers and an increase in the proportion of fast-twitch fibers (IIA, IIB, IIX), whilst preserving the specific force tension. **> **Sham and denervated *RANK*^*mko*^ soleus and EDL muscles exhibited a lower proportion of fast-twitch fibers expressing SERCA-1a and a higher proportion of fast twitch fibers expressing SERCA-2a.
*Genetic deletion of muscle RANK or selective inhibition of RANKL is not as effective as full-length OPG-fc in mitigating muscular dystrophy. Dufresne *et al*., 2018 [40]	*RANK*^*mko*^ mice (specific RANK skeletal muscle deletion) and *mdx* mice were used and cross bred (generating double deficient mice—dystrophin and RANK) *mdx* mice and cross breeds received intraperitoneal injections of: PBS, full-length OPG-Fc, truncated OPG-Fc (1 mg/kg/d), anti-RANKL (1 mg/kg/3d, IK22–5) or anti-TRAIL (1 mg/kg/3d, H2B2). Mice were treated for 10 days (day 25 to 35 post-birth) Functional performance assessment: 5–6-month-old *mdx* mice treated with vehicle (PBS) or full-length OPG-Fc (1 mg/kg/d) for 10 days (prior downhill running)	** > **RANK mRNA was 5.5-fold higher in *mdx* EDL muscles relative to C57BL/6 mice ** > **Compared to PBS treated *mdx* control mice, maximum specific force was significantly higher in *mdx* mice treated with full-length OPG-Fc (soleus, EDL [restored to similar force level of WT] and diaphragm) and *mdx-RANK*^*mko*^ mice (EDL and diaphragm) ** > ***mdx-RANK*^*mko*^ mice treated with full-length OPG-Fc exhibited significantly higher maximum specific force (compared to control); much higher than untreated *mdx-RANK*^*mko*^ mice, indicating OPG may work independently to the RANK-RANKL pathway ** > ***mdx* mice treated with anti-RANKL, anti-TRAIL, combination of anti-RANKL and anti-TRAIL, truncated OPG-Fc (+ 43%) and full-length OPG-Fc all (greatest effect, + 162%) exhibited significantly increased maximum specific force (EDL) ** > **Full-length OPG-Fc treated *mdx* mice were able to travel significantly further pre- and post-eccentric exercise, and significantly more (75%) were able to complete the downhill running protocol (PBS-treated *mdx* mice: 10%) ** > **Full-length OPG-Fc treatment, but not muscle RANK deletion, significantly prevented the loss of force following repetitive eccentric contractions and significantly increased SERCA activity (EDL, over different Ca^2+^ concentrations: pCa 7.6–4.7) in *mdx* mice ** > **Full-length OPG-Fc induced a 6-fold significant increase in EDL SERCA-2a protein levels (but not SERCA-1a) in *mdx* mice, compared to PBS-treated mdx mice ** > **Full-length OPG-Fc treatment rescued maximal ATPase activity in *mdx* EDL muscles
*An anti-RANKL treatment reduces muscle inflammation and dysfunction and strengthens bone in dystrophic mice. Hamoudi *et al*., 2019 [39]	WT and *mdx/utrn*^+*/−*^ mice were treated with 1 or 4 mg/kg anti-mouse RANKL (IK22–5) or PBS. Intraperitoneal injections given every 3 days, from 16 to 20 weeks of age	** > **RANK and RANKL protein levels were higher in *mdx/utrn*^+*/−*^ mice compared to WT ** > **IK22-5 treatment significantly improved the specific force and significantly decreased the muscle mass to body weight ratio of *mdx* EDL and *mdx* soleus muscles compared with PBS-treated *mdx/utrn*^+*/−*^ mice ** > **IK22-5 did not protect *mdx* EDL and *mdx* soleus muscles from repeated eccentric contraction-induced force loss ** > **IK22-5 significantly reduced (35%) serum creatine kinase (muscle damage marker), reduced fibrotic and damaged areas and myofiber regeneration (as shown by a reduction in Pax7^+^ cells and centrally nucleated myofibers) of *mdx* EDL muscles (compared to PBS treated *mdx/utrn*^+*/−*^ mice) ** > **IK22-5 promoted a shift to mid-sized myofibers in *mdx/utrn*^+*/−*^ mice
Testing the efficacy of a human full-length OPG-Fc analog in a severe model of cardiotoxin-induced skeletal muscle injury and repair. Bouredji *et al*., 2021 [37]	Male WT C57BL/10J mice (10–12 weeks old) subject to cardiotoxin (CTX) induced skeletal muscle injury ± intraperitoneal injection of PBS or hFL-OPG-Fc (1 mg/kg/day) for 3 or 7 days	** > **hFL-OPG-Fc treatment significantly improved muscle integrity, fiber CSA, repair and regeneration (as shown by increased central nuclei, centrally nucleated myofiber CSA, Pax7^+^ cells, e-MyHC myofibers, β3 integrin and myogenin) and force of CTX-injured soleus muscles after 7 days
RANKL Mediates Muscle Atrophy and Dysfunction in a Cigarette Smoke-induced Model of Chronic Obstructive Pulmonary Disease. Xiong *et al*., 2021 [38]	Female C57BL/6 mice subject to 24-week cigarette smoke (CS) protocol ± anti-mouse RANKL (100–250 μg/mouse IK22-5) or isotype control antibody (2A3), twice a week from week 1 to week 24	** > **CS significantly increased RANK and RANKL protein levels in the gastrocnemius muscles ** > **CS mice treated with IK22-5 saw significant improvements in body weight, gastrocnemius and soleus weight and myofiber CSA, grip strength, maximal speed, and running time, compared to CS mice ** > **IK22-5 significantly reduced Atrogin-1, MuRF1, myostatin and inflammatory mediators (NF-κB-p65 ratio, TNF-α and IL-6) in the gastrocnemius muscle of CS mice
RANKL Blockade Reduces Cachexia and Bone Loss Induced by Non‐Metastatic Ovarian Cancer in Mice. Pin *et al*., 2022 [34]	8-week-old female NOD-scid/IL2Rgnull (NSG) immunodeficient mice and C26 tumor-bearing 8-week-old CD2F1 male mice were used for cancer related investigations – these mice were given either IK22-5, zoledronic acid, ES-2 cells or C26 cells stably overexpressing RANKL 8-week-old CD2F1 male mice were used for RANKL over-expression (AAV6‐CMV‐RANKL injected into right gastrocnemius)	** > **Elevated RANKL (*via* AAV6‐CMV‐RANKL) induced murine muscle atrophy (reduced muscle mass and CSA), muscle weakness (reduced force) and bone loss ** > **Tumor‐derived RANKL (*via* C26 cells) exacerbated muscle and bone loss in murine cancer cachexia ** > **Anti-RANKL IK22-5 improved muscle mass and strength, preserved bone mass, and normalized RANKL/OPG expression in the mice bearing ovarian cancer ** > **Zoledronic acid improved muscle mass and strength in murine ovarian cancer
Delayed denervation-induced muscle atrophy in Opg knockout mice. Zhang *et al*., 2023 [41]	Assessment of Homozygote *Opg* ^*−/−*^ and WT mice receiving sciatic nerve transection – right hind limb sciatic nerve was exposed and transected, left was just exposed (sham), for 3, 7 and 14 days	** > **Satellite cells isolated from *Opg *^*−/−*^ mice exhibited reduced proliferation but increased differentiation index and fusion index once differentiated for 48 h ** > ***Opg* knockout mice exhibited reduced body mass, but displayed similar functional recovery (e.g., stride length) as WT mice after denervation ** > ***Opg* knockout delayed gastrocnemius muscle atrophy after denervation (3–14 days), as shown by significant increases in gastrocnemius CSA and wet weight ** > **In the *Opg*^*−/−*^ sham group, MuRF-1 and Atrogin-1 was upregulated, compared to WT. > Denervation reduced MuRF1 and increased Atrogin-1 (*Opg*^*−/−*^ gastrocnemius) ** > **Type I (slow-twitch) fibers increased in *Opg *^*−/−*^ mice compared to WT, and *Opg* knockout delayed type IIB myofiber atrophy following denervation ** > **Tet2 expression increased in denervated *Opg *^*−/−*^ mice (gastrocnemius muscle)
*Anti-RANKL Therapy Prevents Glucocorticoid-Induced Bone Loss and Promotes Muscle Function in a Mouse Model of Duchenne Muscular Dystrophy. Jayash *et al*., 2023 [45]	Dystrophic skeletal muscle function and bone microstructure were assessed in *mdx* mice treated with deflazacort (DFZ, 1.2 mg/kg/day) or anti-RANKL (IK22-5, 4 mg/kg/3d), or both for 8 weeks	** > **IK22-5 and DFZ improved grip force performance in *mdx* mice, yet no synergistic effect was observed ** > **While IK22-5 showed enhanced *ex vivo* contractile properties of dystrophic muscles, DFZ did not exhibit the same effect. This improvement in function was correlated with decreased muscle damage, fibrosis, and inflammatory cell count ** > **IK22-5, whether alone or in combination with DFZ, also enhanced the trabecular bone structure in *mdx *mice
RANKL signaling drives skeletal muscle into the oxidative profile. Cavalcanti de Araújo *et al*., 2024 [42]	Male C57BL/6 J (WT) and B6.129S4-Tnfrsf11btm1Eac/J (OPG^−/−^), and OPG^+/−^ mice were used to explore muscle physiology. Skeletal muscle respiratory rate was investigated in WT mice receiving RANKL (1.8 μg/kg/d) for 28 days	** > **Compared to WT and OPG^+/−^ mice, OPG^−/−^ mice exhibited reduced body weight and increased succinate dehydrogenase in the gastrocnemius muscle. OPG^+/−^ and OPG^−/−^ mice exhibited reduced gastrocnemius weight/body weight (%) compared to WT ** > **OPG^−/−^ soleus muscles showed higher oxygen consumption in the states of oxidative phosphorylation, non-phosphorylating, and noncoupled or maximal mitochondrial electron transport state ** > **RANKL treatment to WT mice increased the number of mitochondria per μm^2^, succinate dehydrogenase and muscle force production after a fatigue in the gastrocnemius muscle, and increased oxygen consumption in the states of oxidative phosphorylation and noncoupled mitochondrial electron transport in soleus muscles

Mice over-expressing human RANKL (*huRANKLTg*^+^*)* exhibited significantly lower gastrocnemius and soleus weight (female mice, normalised to body weight), as well as significantly reduced limb muscle force (males only, trend seen in females), skeletal muscle volume, and fine movement versus WT controls. In *huRANKLTg*^+^ mice, denosumab increased gastrocnemius and soleus weight and maximal speed, whereas OPG-Fc increased soleus weight only. In a mouse model of osteosarcopenia and impaired glucose homeostasis (*Pparb*^*−/−*^), OPG-Fc significantly increased maximum force of the limb (normalised to gastrocnemius mass), skeletal muscle volume, and area and number of type I (slow-twitch) fibers, restoring parameters to that of WT mice [22] (Table 3).

A cardiotoxin-induced (CTX) skeletal muscle injury model explored the efficacy of human full-length (hFL)-OPG-Fc in aiding skeletal muscle repair. Seven days post injury, hFL-OPG-Fc treatment significantly improved isometric and specific force of the soleus muscle, but had no effect on soleus muscle mass [37]. In a cigarette smoke (CS)-induced model of chronic obstructive pulmonary disease (COPD) in mice, IK22-5 treatment significantly improved body weight, gastrocnemius and soleus weight, grip strength, running time and maximal speed to values close to those seen in control (air) mice [38]. The role of enhanced RANK-RANKL signalling in reducing muscle mass and function is supported by a reversal of effects with known RANKL inhibitors, hFL-OPG-Fc [37] and IK22-5 [38]. In parallel, IK22-5 treatment to *mdx/utrn*^+*/−*^ mice, also improved the specific force of EDL and soleus muscles (versus PBS-treated *mdx/utrn*^+*/−*^ mice) and dampened the effects of edema [39] (Table 3).

Injection of anti-TRAIL, IK22-5, truncated OPG-Fc (RANKL-binding domain only) and full-length OPG-Fc into *mdx* mice significantly increased EDL specific force by 17%, 45%, 43% and 162% respectively, versus *mdx* control. Full-length OPG-Fc also increased maximum specific force of *mdx* soleus and diaphragm muscles. *mdx-RANK*^*mko*^ mice exhibited significantly increased specific force of EDL, soleus and diaphragm muscles (versus *mdx* control). Moreover, full-length OPG-Fc treatment of *mdx-RANK*^*mko*^ mice further enhanced the increase in EDL force compared with *mdx* and *mdx-RANK*^*mko*^ groups, thereby suggesting OPG-Fc may benefit dystrophic muscle independent of RANK-RANKL [40] (Table 3).

Despite these reports of efficacy of OPG treatment and/or other means of RANKL inhibition (e.g., IK22-5) in both dystrophic and non-dystrophic models, uncertainties remain due to disparities between studies (e.g., muscles analysed and treatment schedules), and in some cases only marginal improvements in test parameters. It is difficult to conclusively state that direct inhibition of RANKL in skeletal muscle may benefit its mass and function in clinical settings. Furthermore, two recent observations demonstrate a beneficial effect of RANKL signalling on skeletal muscle physiology: OPG knockout delays rather than accelerates gastrocnemius muscle atrophy after denervation in mice [41], and RANKL signalling in skeletal muscle (*in vitro* and *in vivo*) promotes mitochondrial biogenesis and subsequently benefits the oxidative profile of the muscle [42]. These observations are inconsistent with the hypothesis that OPG activity and/or RANKL inhibition is inherently beneficial to skeletal muscle (Table 3). Rather, the complexity of the RANK-RANKL-OPG axis in skeletal muscle physiology requires further study to understand and develop potential therapeutic strategies.

## Potential Mechanisms of RANK-RANKL-OPG Axis in Skeletal Muscle

### Skeletal Muscle Regeneration

**Fig. 1:**
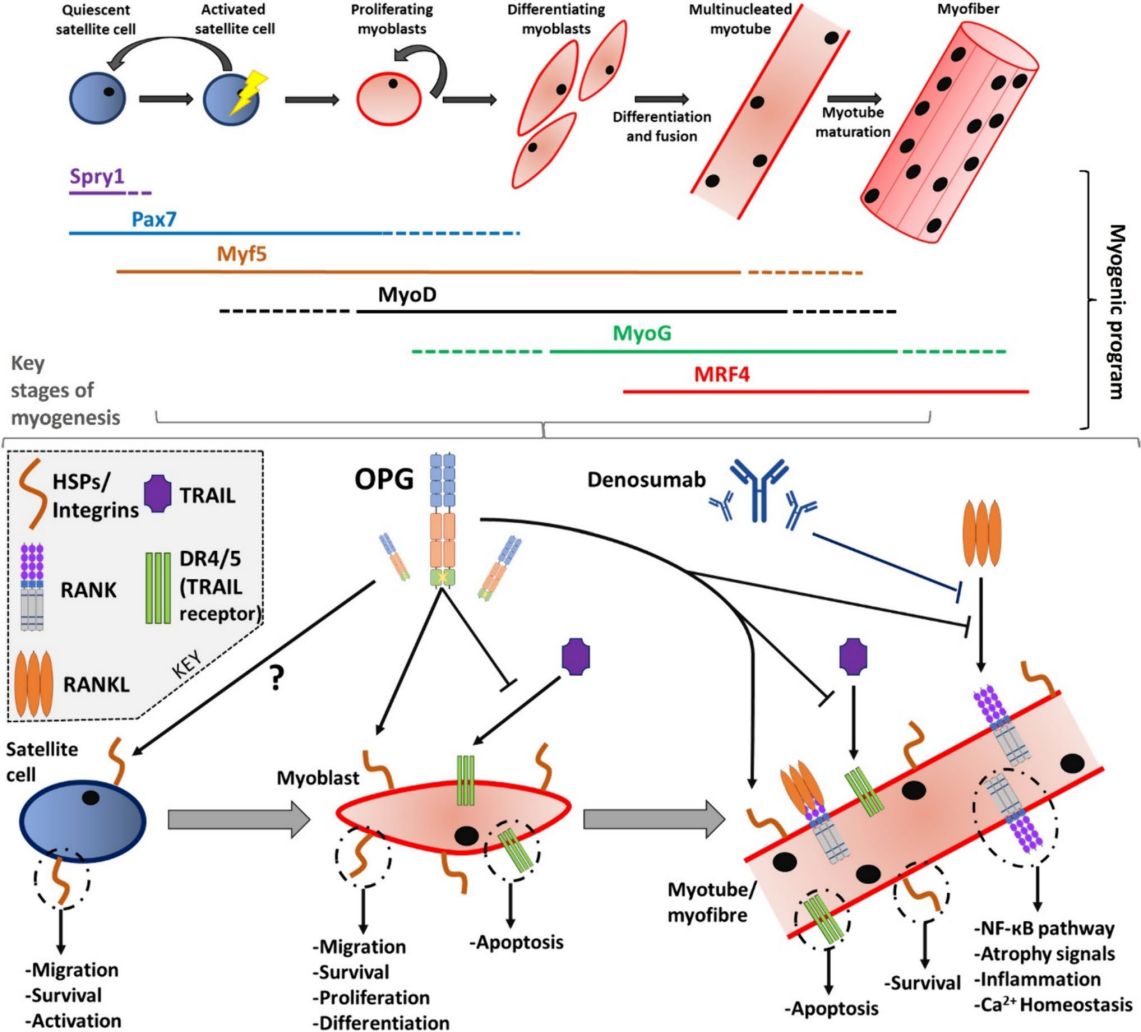
The myogenic program of skeletal muscle (top) and potential points during myogenesis in which factors of the RANK-RANKL-OPG axis may affect skeletal muscle physiology (bottom). RANK-RANKL signalling may have the greatest effect on differentiated myoblasts, i.e., myotubes, as growing evidence suggests RANK expression is confined to myotubes. OPG may influence all stages of muscle regeneration, particularly earlier stages through its ability to bind to TRAIL, heparan sulfate proteoglycan (HSPs) and integrins. RANK-RANKL signalling may be a regulator of NF-κB dynamics, atrophy networks, inflammatory signals and Ca^2+^ homeostasis in skeletal muscle

Muscle regeneration is a complex process, one that involves numerous stages of muscle stem (satellite) cell activation, proliferation, differentiation, and maturation, collectively called myogenesis (Fig. 1).

Ageing and disease can disrupt myogenesis, leading to reduced muscle mass and function [46]. On the contrary, an increase in muscle stem cell activity and muscle regeneration can result in an improvements in muscle mass and function [47]. The improvements seen in muscle mass and function following disruption of the RANK-RANKL pathway indicate a potential beneficial effect on myogenic mechanisms. However, currently, evidence of this is lacking and more exploration is required.

As myogenesis constitutes different stages, it is important to consider the expression profile of the RANK-RANKL-OPG axis throughout myogenesis. RANK is expressed in C2C12 myotubes [36], which was corroborated in primary mouse and immortalised (KM670) human myotubes [48]. RANKL was shown to be present at low concentrations in C2C12 myotubes [34], and in human KM670 myotubes [48]. OPG is released from C2C12 myotubes [15]. As well, it was shown that OPG is secreted from KM670 myoblasts and OPG release increases as myogenic differentiation initiates and progresses [48]. These observations indicate that the RANK-RANKL signalling may have a more dynamic affect in the later stages of myogenesis (Table 2), whereas OPG may regulate and influence earlier myogenic stages, by virtue of its heparin binding and death domains (Fig. 1).

**Fig. 2:**
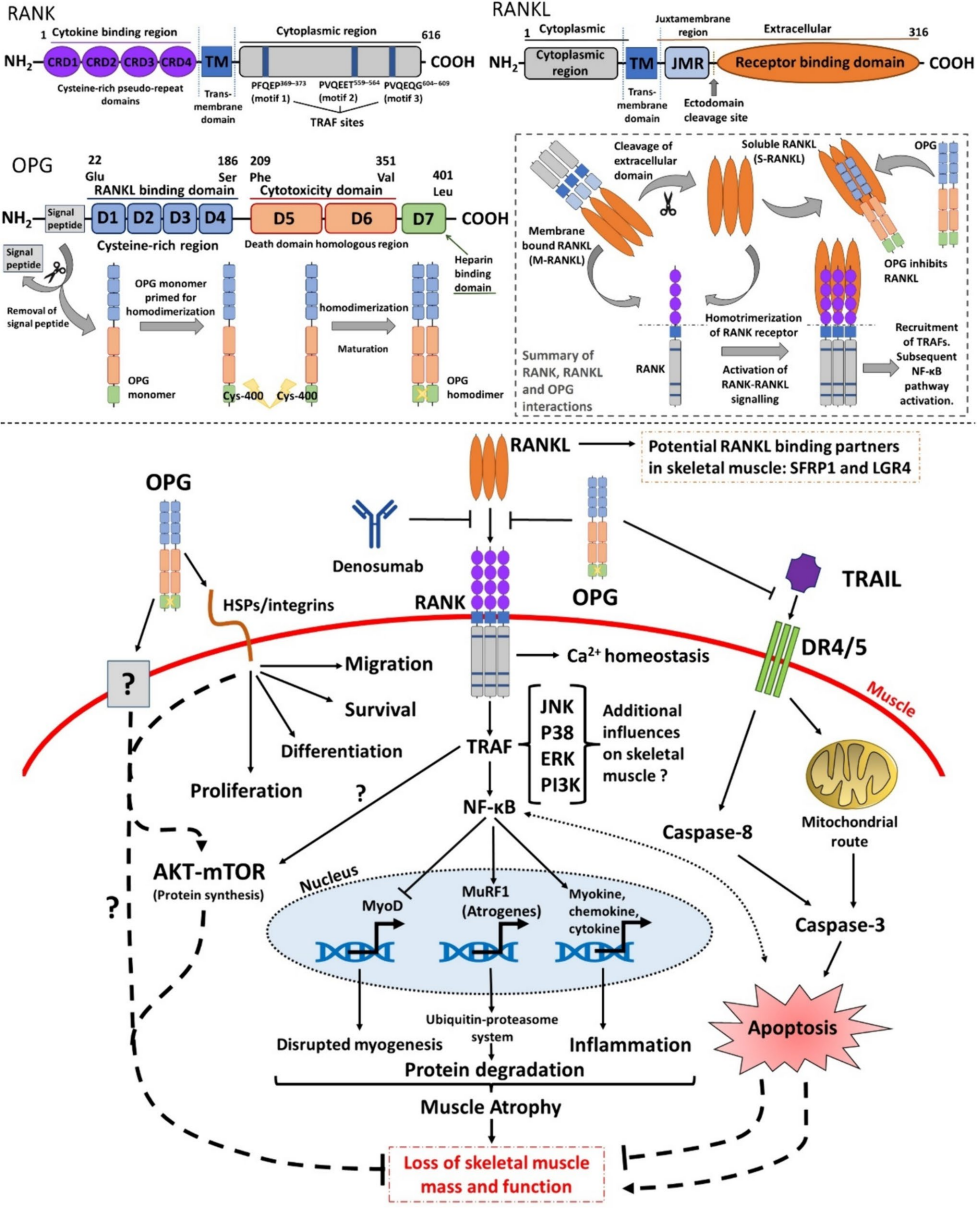
The OPG-RANKL-RANK in skeletal muscle – potential influences of RANKL inhibitors on sarcopenia. Top: domain structures of RANK, RANKL and OPG (inclusive of homodimerization activation stage) and general overview of RANK-RANKL-OPG signalling. Bottom: Summary of potential ways in which RANKL inhibitors may modulate skeletal muscle atrophy and subsequently sarcopenia. As well as inhibiting RANKL, OPG may modulate apoptosis pathways *via* interactions with TRAIL and may elicit responses in muscle *via* cooperation with surface proteins (e.g. integrins). TRAF may influence AKT dynamics in muscle [66, 67]. There may exist some other unknown modes of actions of OPG in skeletal muscle. Dashed lines represent potential RANK-RANKL independent interactions. RANKL may also interact with SFRP1 [68] and LGR4 [69] in skeletal muscle

In a mouse model of CTX-induced skeletal muscle injury, hFL-OPG-Fc treatment increased two key markers of myogenesis, centrally nucleated cells and embryonic myosin heavy chain (eMyHC) myofibers, as well as satellite cell density as represented by Pax7^+^ cells, myogenin expression (potent mediator of myogenic differentiation) and β3 integrin expression [37]. An increase in eMyHC expression correlated with an increase in contractile tissue, indicating improved regeneration [37]. In parallel, C2C12 myotubes treated with RANKL exhibited reduced eMyHC expression [38], highlighting the RANK-RANKL-OPG axis may influence eMyHC expression and subsequently regenerative capacity. Bouredji *et al*. suggest that the effects of hFL-OPG-Fc on muscle may be initiated through integrin signalling as OPG has a heparin binding domain (Table 3) [37]. OPG interaction with these cell surface molecules (e.g., integrins and HSPs, Fig. 1–2) has been shown to promote proliferation, survival and migration [49]. Furthermore, the expression of HSPs [50] and β3 integrins [51] are increased during skeletal muscle regeneration, thereby suggesting OPG may potentially work in a dualistic fashion in skeletal muscle, binding to RANKL and HSPs/integrins to aid regeneration. *In vitro*, hFL-OPG-Fc treatment significantly increased C2C12 myotube fiber diameter and fusion index, both of which reflect a more pro-myogenic phenotype [37]. Conversely, RANKL treatment of C2C12 cells was associated with significantly lower myotube cross sectional area (CSA) [35]. Taken collectively, these findings suggest RANKL signalling is potentially deleterious to myogenic potential *in vitro* (Table 2).

Interpretation of potential mechanistic effects is, however, complex and differs between models. An example is that RANK and RANKL protein levels are elevated in the EDL of *mdx/utrn*^+*/−*^ mice compared with WT mice. In contrast to the CTX model above, following IK22-5 anti-RANKL treatment, the number of satellite cells (Pax7^+^ cells) and the proportion of centrally nucleated myofibers was lower [39] (Table 3). This apparent paradox arises because satellite cell hyperplasia due to asymmetric cell division dysfunction is a common manifestation in muscular dystrophy resulting in mitotic defects and reduced numbers of myogenic progenitors [52]. Hence, the observations here suggest asymmetric and symmetric satellite cell division is returning to a more balanced state, one which favours efficient muscle regeneration and tissue homeostasis. Further research is warranted regarding the potential interplay of the RANK-RANKL-OPG axis and the myogenic program (Fig. 1) in both healthy and pathological muscle.

### Changes in Muscle Fiber Morphology and Contractile Properties when the RANK-RANKL Pathway is Dysregulated

OPG and RANKL inhibition has been shown to benefit the morphology of myofibers, mostly favouring a more pro-myogenic state. For example, OPG knock out mice exhibited reduced CSA of both EDL myofiber and fast-twitch-type IIb fibers [35] (Table 3), while hFL-OPG-Fc treatment of C2C12 cells *in vitro* enhanced the diameter of myotubes, from which new myofibers are derived [37] (Table 2). Increased primary mouse myotube diameter following OPG treatment has also been observed in a different investigation [48]. hFL-OPG-Fc treatment to CTX muscle injury also significantly increased the CSA of soleus muscle fibers, as well as that of centrally nucleated myofibers, 7 days after injury [37]. IK22-5 improved the myofiber CSA of gastrocnemius and soleus muscles in a CS-induced model of COPD [38]. In *mdx/utrn*^+*/−*^ mice, IK22-5 treatment promoted a favourable shift in myofiber size, with CSA distribution frequency revealing a ~ 3-fold reduction in the number of small myofibers (< 500 μm^2^) accompanied by a significant increase in the number of intermediate-sized myofibers (2500 μm^2^) [39] (Table 3).

Emerging evidence suggests that the fiber composition of skeletal muscle plays a part in the responsiveness to alterations in the RANK-RANKL-OPG axis [35], and the axis may potentially promote fiber-type switching e.g., in *Opg*^*−/−*^ mice, a type I (slow-twitch) to type II (fast-twitch) switch was observed [41], whereas in *huRANKLTg*^+^ mice, denosumab treatment switched type II fibers to type I [22] (Table 3). Muscles comprising predominantly fast-twitch fibers (e.g. EDL) display a greater response to changes in the RANK-RANKL pathway. For example, at 5 months, *Opg*^*−/−*^ mice exhibited significantly reduced contractile properties of the EDL, whereas, contractile properties of the predominately slow-twitch soleus muscles were not significantly affected [35]. Type I fibers were almost absent in *RANK*^*mko*^ EDL muscles, versus *RANK*^*floxed/floxed*^ EDL. In denervated *RANK*^*mko*^ EDL muscles, the proportion of fast-twitch fibers, specifically IIA, IIB and IIX, were significantly increased compared to denervated *RANK*^*floxed/floxed*^ EDL. RANK ablation was shown to preserve the specific force tension of denervated EDL, but this effect was not mimicked in slow-twitch soleus muscles [36] (Table 3).

### Calcium-mediated Effects

The rapid, beneficial effects of denosumab/RANKL inhibition on dystrophic symptoms in a case study of facioscapulohumeral muscular dystrophy are unambiguous and have not been reported in any other clinical cases (Table 1). The change in medication the patient received (teriparatide to denosumab) may predicate the potential improvements seen here [24]. Teriparatide is pro-calcemic, whereas denosumab is anti-calcemic [53]. Impaired Ca^2+^ homeostasis underpins multiple dystrophic abnormalities [54]. Hence, in this case, the rapid improvements in dystrophic symptoms could be attributed to potential alterations in Ca^2+^ flux in the dystrophic muscles or in circulation. Investigating denosumab (acute and rapid blood Ca^2+^ reduction) against bisphosphonates (slower and adaptive blood Ca^2+^ reduction) may provide new answers regarding the potential association between RANKL inhibition and Ca^2+^ signalling in ameliorating skeletal muscle dysfunction.

Preclinical work has explored some potential links between the RANK-RANKL-OPG axis and Ca^2+^ homeostasis in skeletal muscle. Both sham and denervated *RANK*^*mko*^ soleus and EDL muscles exhibited a lower proportion of fast-twitch fibers expressing sarco/endoplasmic reticulum Ca^2+^-ATPase (SERCA)-1a and a higher proportion of fast twitch fibers expressing SERCA-2a. These results suggest RANK is a key regulator of SERCA activity and Ca^2+^ storage [36]. SERCA regulates Ca^2+^ transport from the cytosol into the sarcoplasmic reticulum and is important in facilitating muscular relaxation post contraction. SERCA-1a is expressed in fast-twitch skeletal muscle fibers, whereas SERCA-2a is limited to cardiac and skeletal muscle slow-twitch muscle fibers [55] (Table 3). It appears that RANKL signalling has a more noticeable negative effect on fast-twitch fibers, and its inhibition can potentially influence SERCA activity, Ca^2+^ flux, and fiber-type switching.

Full-length OPG-Fc has shown to significantly increase SERCA activity, as measured by ATPase in *mdx* EDL muscles, over different Ca^2+^ concentrations. Muscle RANK deletion did not influence SERCA activity in this *mdx* model. Moreover, full-length OPG-Fc induced a 6-fold significant increase in *mdx* EDL SERCA-2a protein levels, but not SERCA-1a [40] (Table 3). Alterations to the RANK-RANKL-OPG axis induce similar effects on SERCA-1a/2a, in both dystrophic and non-dystrophic muscle, therefore suggesting a common modality of the RANK-RANKL-OPG axis in skeletal muscle may be Ca^2+^ regulation, which is vastly important in the context of skeletal muscle homeostasis [36].

### Markers of Inflammation and Skeletal Muscle Atrophy

The ubiquitin proteasome system has an influential role on skeletal muscle protein degradation, subsequently regulating atrophy/hypertrophy pathways. Dysfunction of this system promotes muscle wasting as seen in age-associated sarcopenia. Atrogin-1 and MuRF-1 are two E3 ubiquitin ligases which are defined as major regulators of ubiquitin-mediated protein degradation in skeletal muscle [56]. Furthermore, as well as regulating inflammation, NF-κB signalling (a pivotal target of RANKL-RANK) plays an influential role in skeletal muscle atrophy [57]. Collectively, Atrogin-1, MuRF-1 and NF-κB are considered key players in progressive muscle wasting (Fig. 2).

In the fast-twitch-dominant tibialis anterior (TA) muscle of *Opg*^*−/−*^ mice, MuRF-1 and Atrogin-1 protein levels were significantly elevated (1.6- and 2-fold higher, respectively) compared to WT mice. The ratio of active p-NF-κB-p65/NF-κB-p65 was also significantly higher in *Opg*^*−/−*^ mice (Table 3). The same authors recorded significant increases in Atrogin-1, MuRF-1 and p-NF-κB-p65 (Ser536) protein levels in C2C12 myotubes treated with RANKL [35], similar effects were also seen in [38] (Table 2). siRNA-RANK in combination with RANKL treatment significantly reduced these atrophy markers compared to C2C12s treated solely with RANKL [38] (Table 2). Interestingly, anti-RANKL (IK22-5) treatment was shown to significantly reduce MuRF-1, Atrogin-1 and myostatin (a potent negative regulator of skeletal muscle growth) protein levels in the gastrocnemius muscle of mice exposed to CS (COPD model). IK22-5 treatment attenuated inflammation, as shown by a reduction of p-NF-κB-p65(Ser536)/NF-κB-p65 ratio, TNF-α and IL-6, in skeletal muscles from CS-exposed mice [38] (Table 3). Contrasting with this, a recent publication showed a reduction in MuRF1 (and PTPRG, an anti-myogenic protein) expression in C2C12 myotubes after 24 h of RANKL treatment [42], highlighting the puzzling mechanisms of RANKL signalling in skeletal muscle (Table 2).

OPG-Fc reversed the effects of chronic RANKL exposure on C2C12 myotubes by blocking NF-κB signalling, as shown by decreased mRNA expression of Fos, Jun and NFAT, whilst inducing no effect on apoptotic markers (e.g. Bcl2) or Ca^2+^ signalling (e.g. Syk). Furthermore, in *Pparb*^*−/−*^ mice, OPG-Fc treatment significantly reduced genes related to NF-κB signalling (e.g. NFAT and Jun) [22] (Table 3). It is important to mention here that components and downstream targets of RANKL mediated NF-κB signalling may also be implicated in skeletal muscle atrophy and homeostasis. TRAF6, an E3 ubiquitin ligase, is indispensable during RANK signal transduction and can also interact with p62/SQSTM1 [58]. Loss of TRAF6 activity has been associated with reduced muscle atrophy *in vivo* and *in vitro* [59–61]. P62/SQSTM1, a classical selective autophagy receptor, also plays a part in the ubiquitin–proteasome system. P62/SQSTM1 has shown to accumulate in sarcopenic [62] and aged [63] skeletal muscle. Hence, RANK-TRAF6-p62/SQSTM1 interactions may provide mechanistic explanation to some published findings and warrants further exploration. Moreover, the NFAT family of Ca^2+^-dependent transcription factors, particularly NFATc1, is a target of RANKL mediated NF-κB signalling. NFATc1 activity controls fiber type and is a negative regulator of MyoD (Fig. 1) activity in skeletal muscle [64]. Interplay between NFAT activity and Ca^2+^ flux may influence skeletal muscle homeostasis. These points add to the complexity and ambiguity of the potential roles of the RANK-RANKL-OPG axis in skeletal muscle physiology and atrophy dynamics (Fig. 2).

Dystrophic *mdx/utrn*^+*/−*^ mice exhibit excessive fibrosis and inflammation compared to other mdx mouse models. IK22–5 treatment to *mdx/utrn*^+*/−*^ mice has been shown to significantly reduce serum creatine kinase (35%), areas of fibrotic damage in the EDL muscle, the number of pro-inflammatory M1 macrophages (38%) and increase anti-inflammatory M2 macrophages (132%) [39]. As well as regulating inflammatory responses, M2 macrophages are also involved in muscle growth and regeneration [65]. Hamoudi *et al.* observed elevated levels of M2 macrophages following IK22-5 treatment to *mdx/utrn*^+*/−*^ mice, indicating potentially improved muscle regeneration support. IK22–5 significantly reduced the p-NF-κB-p65(Ser536)/NF-κB-p65 ratio in the TA muscle of *mdx/utrn*^+*/−*^ mice, suggesting RANKL signalling in dystrophic muscle promotes inflammation and its inhibition reduces this [39]. Taken collectively, growing evidence suggests the RANK-RANKL-OPG axis may influence inflammatory and atrophic pathways, which is also reflected phenotypically to a degree (refer to Tables 2 and 3).

## The Effects of OPG and the RANK-RANKL Pathway in Cardiac Muscle

A review by Marcadet *et al.* highlights potential roles of the RANK-RANKL-OPG axis in cardiac muscle [70]. Here we add to their discussion. Like skeletal muscle, cardiac muscle is striated and composed of repeated sarcomeres. Cardiomyocytes, the active cellular unit of cardiac muscle, function as a syncytium, contract involuntarily and exhibit branched fibers [71]. Human recombinant OPG significantly promoted cardiomyocyte hypertrophy and inhibited autophagy, as shown by a significant reduction in LC3-II expression (autophagosome formation marker) and increased p62/SQSTM1 in H9C2 cardiomyocytes. It was also shown that OPG (5 mg/kg/day) treatment for 7 days significantly stimulated cardiac hypertrophy (as shown by heart/body weight ratio) in 14-week-old mice. Furthermore, the hypertrophic effects of OPG were exacerbated by 3-Methyladenine (autophagy inhibitor) [72]. Contrary to OPG treated young mice, aged *OPG*^*−/−*^ mice exhibit increased heart and left ventricle (LV) weight, apoptotic cells and TRAIL activation, as well as reduced cardiac wall thickness and contractile function. OPG treatment to *OPG*^*−/−*^ mice partially improved LV structure and function suggesting OPG may play a role in preserving myocardial structure and function during aging [73]. Clinically, OPG levels positively correlated with LV wall thickness, with authors speculating OPG, *via* potential interactions with the renin-angiotensin system, promotes cardiac hypertrophy [74]. Recently, Marcadet *et al.* showed anti-RANKL IK22-5 treatment significantly reduced heart mass and LV hypertrophy, maintained cardiac function, and inhibited two mediators of cardiac hypertrophy, NF-κB and PI3K in *mdx* mice (versus *mdx* controls). As well, anti-RANKL treatment potentially improved Ca^2+^ homeostasis in dystrophic hearts as shown by increased SERCA activity and increased SERCA2a, RyR and FKBP12 expression [75].

## The Role of the OPG-RANKL-RANK *Axis* in Neural Tissue

Components of OPG-RANKL-RANK axis have been shown to be expressed in prominent cell types within neural tissue [7, 8], including microglia [76, 77]. OPG has also been shown to be highly expressed in spinal cord and cerebrospinal fluid and positively correlates with age in patients with non-inflammatory neurological diseases [78]. It has been shown that microglia express Toll-like receptors (TLRs). RANKL-RANK signalling has the capacity to decrease TLR3/TLR4 mediated expression of inflammatory markers e.g., iNOS and cyclo-oxygenase 2. Conversely, TLR4 supresses RANK expression and simultaneously enhances TLR3, which intensifies pro-inflammatory signalling *via* a positive feedback loop. Adding to this, OPG may influence this pathway and may block pro-apoptotic TRAIL [77, 79]. An important study by Hanada *et al.* found that RANKL-RANK signalling was influential on the central fever response in inflammation and female thermoregulation in mice [8]. In rodents, RANKL intracerebroventricular injections triggered a severe fever (whereas intraperitoneal RANKL injections did not) and OPG alleviated the fever. Tissue-specific Nestin-Cre and GFAP-Cre *rank*^*floxed*^ deleter mice revealed RANK was implicated in the fever response and was genetically mapped to astrocytes. RANKL induced fever *via* the COX2-PGE2/EP3R pathway and activated brain regions involved in thermoregulation. Furthermore, impaired fever during pneumonia was observed in two children with RANK mutations [8]. As thermogenesis is metabolically demanding, the RANK-RANKL-OPG axis could be associated with energy supply and subsequently glucose metabolism. Hence, the RANK-RANKL-OPG axis could have additional roles in metabolically demanding tissues (e.g., muscle and liver) and conditions associated with altered glucose metabolism (e.g., diabetes mellitus, DM).

## RANKL Inhibition, a Novel Target to Treat Diabetes Mellitus?

Patients with Type 2 DM exhibit higher serum OPG levels [80]. Conversely, in patients with Type 1 DM, plasma OPG levels were significantly lower and serum RANKL levels were significantly higher compared to controls [81]. Moreover, OPG is a useful biomarker in predicting loss of glycaemic control and associated deterioration of albuminuria [82]. Recently, in a large-scale analysis, it was reported that denosumab treatment was associated with a lower risk of diabetes incidence in patients with osteoporosis [83].

The RANK-RANKL-OPG axis is expressed in and/or regulates prominent tissues associated with glucose metabolism, including pancreatic islet cells [84], skeletal muscle [22], fat/adipocytes [85] and hepatic tissue/hepatocytes [86]. Accumulatively, experimental investigations suggest RANKL signalling is deleterious to glucose homeostasis, with RANKL signalling increasing skeletal muscle [22] and hepatic [87, 88] insulin resistance, *via* NF-κB activation. It has been speculated that RANKL could induce NF-κB-inducing kinase, subsequently impairing glucose-stimulated insulin secretion in human islets [89]. However, *in vivo* (OPG^− / −^ mice and WT mice infused with RANKL) and *in vitro* (preadipocyte lineage 3T3-L1) investigations showed the capacity of RANKL to potentiate beige adipocyte differentiation. This is important in the context of DM as beige adipocytes improve insulin responsiveness and lipid and glucose metabolism. OPG deficiency also improved glucose metabolism when subject to a high-fat diet. Collectively, it is important to note tissue specific effects of RANKL signalling on regulating glucose metabolism [85]. Bonnet *et al.* showed *huRANKLTg*^+^ mice exhibit decreased glucose uptake and increased PTP-RG expression in skeletal muscle, which is associated with inflammation-induced insulin resistance (Table 3). Furthermore, prolonged RANKL exposure in C2C12s increased phosphorylation of IRS1(ser318), which is known to downregulate the activity of insulin receptor 1. This affect was reversed by OPG-Fc treatment [22] (Table 2). As well, RANKL signalling has been shown to reduce human and rodent pancreatic β-cell proliferation. Both OPG and denosumab showed to promote β-cell replication – mechanistically it was shown that inhibition of RANKL modulated CREB and GSK3 pathways [84]. More recently, it was shown that RANK-TRAF6 interactions and subsequent NF-κB activation mediates cytokine-induced rodent and human β-cell death. Both denosumab and OPG provided cytotoxicity protection. In nonobese diabetic/Ltj mice, OPG reduced insulitis, reversed recent-onset T1DM, increased β-cell mass and proliferation, as well as plasma insulin and improved glucose homeostasis. Moreover, denosumab and OPG reduced human T1DM serum–induced β-cell cytotoxicity and dysfunction [90].

## Conclusion and Future Directions

The RANK-RANKL-OPG axis is a pivotal regulator of bone homeostasis, and emerging evidence suggests this axis also plays a role in extra-osseous tissues, with recent publications suggesting functional involvement in skeletal muscle homeostasis and pathophysiology. The beneficial effects of RANKL inhibition on skeletal muscle function and mass in humans and *in vivo,* and *in vitro* models are promising observations. However, the exact mechanistic actions of OPG and other means of RANKL inhibition in skeletal muscle is poorly understood. Adding to this, disparities between different studies highlight the potential complexity of the RANK-RANKL-OPG axis in normal, pathological, and aged skeletal muscle. For example, there lacks robustness between improvements in falls and muscle parameters in PMO women and analogous rodent models [16, 22, 23]. As well, varying effects on atrophy networks in OPG knockout mouse models indicate the enigmatic function of the RANK-RANKL-OPG axis in skeletal muscle [35, 41].

There is an urgent need for new pharmaceutical treatments to ameliorate muscle wasting during ageing and in disease e.g., sarcopenia, cachexia, muscular dystrophies, and certain myopathies. We encourage further research on the roles of the RANKL-RANK-OPG axis in skeletal muscle, particularly on Ca^2+^ homeostasis, NF-κB dynamics, myogenic potential and atrophy mechanisms (e.g., TRAF6, p62/SQSTM1 and MuRF1). Utilising different OPG constructs (e.g. truncated forms vs full-length) in conjunction with and compared to RANKL inhibitors will help to further elucidate the exact mechanistic actions in muscle. Increased knowledge of the RANKL-RANK-OPG axis in muscle may provide novel solutions to improve muscle function and mass, frailty parameters and/or may contribute to new knowledge regarding skeletal muscle physiology. Finally, dysregulation of the RANK-RANKL-OPG axis appears to occur within ageing tissues, especially bone [91]. More research is warranted to understand how the RANK-RANKL-OPG axis influences neural and homeostatic regulatory tissues, especially glucose metabolising organs including muscle, adipose, the pancreas and the liver, during ageing.

## Data Availability

No datasets were generated or analysed during the current study.
